# Perioperative analgesic effects of a modified supratemporal retrobulbar block in dogs undergoing corneal and endocular surgery

**DOI:** 10.1111/jsap.13829

**Published:** 2025-01-12

**Authors:** E. Lardone, M. Crasta, P. C. Ostan, P. Gherlinzoni, A. Landi, P. Franci

**Affiliations:** ^1^ Department of Veterinary Science University of Turin Grugliasco Italy; ^2^ AniCura VisionVet Bologna Italy

## Abstract

**Objectives:**

To evaluate the perioperative efficacy of a modified supratemporal retrobulbar block in dogs undergoing ocular surgery.

**Materials and Methods:**

In this prospective randomised clinical trial, dogs were premedicated with dexmedetomidine (1 mcg/kg im) and methadone (0.1 mg/kg im), induced with propofol to effect and maintained with isoflurane (FE'Iso 1.1%). In the retrobulbar group a mixture of lidocaine 2% (5.5 mL) and ropivacaine 0.75% (2 mL) was administered at 0.1 mL/kg, via a modified supratemporal technique using a Tuohy needle. No block was performed in the controls. When heart rate or mean arterial pressure increased above 30% of the pre‐incisional values, fentanyl (1 mcg/kg iv) was administered. Propofol (1 mg/kg iv) was injected when anaesthesia was deemed too light. After a total of three administrations regardless of the type of drugs (fentanyl/propofol), a constant rate infusion of fentanyl (5 mcg/kg/h iv) was started. Quality of recovery (blindly assessed using a descriptive score scale), postoperative eye rubbing and complications were studied.

**Results:**

Eighteen dogs were included. The retrobulbar group (nine) dogs had significantly less risk of receiving fentanyl than controls (nine) (Relative risk:  0.142, 95% CI: 0.021 to 0.936) and a recovery score > 2 (RR: 0.058, 95% CI: 0.003 to 0.887). The median amount of fentanyl (mcg/kg) was statistically lower in the retrobulbar group than in the controls: 0 mcg/kg (range, 0 to 1) versus 2 mcg/kg (range, 0 to 8.49), respectively. Only controls showed eye rubbing.

**Clinical Significance:**

The modified supratemporal retrobulbar block reduced the intraoperative rescue analgesia and improved the quality of recovery.

## INTRODUCTION

The retrobulbar block entails the deposition of a local anaesthetic within the ocular cone. This anaesthesia technique was first described by Herman Knapp in 1884 (Polania Gutierrez & Riveros Perez, 2023) and is still routinely used today because of its ease to produce analgesia, immobilisation and protrusion of the eye in corneal and intraocular surgeries (Simonson, 1990).

In their study in dogs, Accola et al. (2006) compared three techniques used in veterinary medicine to perform RB or peribulbar block: the inferior‐temporal lid injection approach, the perimandibular approach and the combined superior–inferior peribulbar approach. The inferior‐temporal palpebral approach (ITP) proved the best of the three because it was effective, easy to perform and provided complete intraconal coverage without complications. Furthermore, the technique produced pupil dilatation and globe centralisation in all eyes.

In clinical practice, the RB is commonly performed in animals undergoing enucleation though it may not provide complete antinociception for surgery on extraconal and extrabulbar tissues. In enucleations, the RB is inadequate for blocking part of the stimuli emerging from the surgical field (Scott et al., 2012). It is a well‐established fact that retrobulbar anaesthesia does not provide complete anaesthesia of the eyelids. This is because branches of the maxillary trigeminal nerve pass external to the extraocular muscle cone (Shilo‐Benjamini, 2019). To date, only one study has investigated the use of RB in ocular surgery different than enucleation, testing the use of the inferior palpebral technique for phacoemulsification in dogs (Hazra et al., 2008). More studies need investigating the potential advantages of the RB in dogs undergoing ocular surgery.

First described by Chiavaccini et al. (2017) in canine cadavers and compared to the ITP approach, the supratemporal approach ensured diffusion of contrast medium comparable to the ITP approach and better distribution of the solution towards the orbital fissure, where the ophthalmic nerves exit the skull. Since the orbital cavity is anatomically open frontally and laterally, the needle can be inserted via the large lateral opening in small animals, as described by Chiavaccini et al. (2017). In this approach, needle insertion is placed farther from the bulbar globe than in frontal techniques. Also, the length of the orbital cone and its lateral surface facilitate hitting the correct target. These differences may reduce the risk of accidental puncture of the ocular bulb when performing the block.

### Scoping search of the literature

The following databases (PubMed, Google Scholar and Web of Science) have been searched on July 12, 2024 using the following keywords: RB, dog, cat, enucleation, corneal surgery, endocular surgery, analgesia. No other reports of RB have been found using these search terms.

The aim of this study was to investigate the intraoperative analgesia provided by the RB via the supratemporal approach (Chiavaccini et al., 2017) in dogs undergoing corneal and intraocular surgeries, compared to a control group. Our hypothesis was that the RB would provide a better intraoperative analgesic‐sparing effect than controls. The null hypothesis was that there was no difference in the intraoperative rescue analgesia between groups.

## MATERIALS AND METHODS

This randomised clinical trial was approved by the Bioethics Committee of the University of Turin, Italy (no. 0421477 – July 29, 2022). The study was performed at the University of Turin, Italy between August 2022 and January 2023. Written informed consent was obtained from all dog owners.

### Animals

Inclusion criteria were scheduled intraocular and corneal surgery, American Society of Anaesthesiologists (ASA) physical status I‐III. Exclusion criteria were aggressive behaviour, severe heart disease and eye surgery other than corneal or intraocular procedures (*e.g*. eyelid surgery). Preoperative clinical examination and complete blood count and serum biochemistry were performed in all animals.

### Outcomes

The primary endpoint was intraoperative analgesia, which was defined prospectively in the protocol. The secondary endpoints, also defined prospectively in the protocol, were intraoperative propofol consumption, recovery quality and description of the RB (in terms of failure rate and time to perform the technique).

### Randomization

Dogs were randomly assigned following simple randomisation procedures (based on a computer‐generated randomisation sequence – www.randomizer.org – without stratification) to one of two treatment groups: retrobulbar group (RG, receiving RB) or control group (C, no RB).

All animals were administered intramuscular (im) methadone 0.1 mg/kg (semfortan; 10 mg/mL, Dechra Veterinary Products, UK) and dexmedetomidine 1 mcg/kg (dexdomitor; 0.5 mg/mL, Orion Pharma, Finland) at the quadriceps muscle. Once sedated, an intravenous (iv) catheter (Delta Med, Italy) was placed in the cephalic vein and anaesthesia was induced with iv propofol (Proposure; 10 mg/mL, Merial, Italy). The animals were then intubated and connected to a circle breathing system. Anaesthesia was maintained with isoflurane (isoflo; Zoetis, Italy) with a targeted end‐expiratory fraction (FE'Iso) of 1.1% in a mixture of oxygen and air (inspired fraction O₂ 0.4%). Lactate Ringer's solution was administered iv at 5 mL/kg/h (Lactated Ringer, Fresenius Kabi, Italy) from intubation to completion of surgery. Volume‐controlled mechanical ventilation was set to maintain an end‐tidal CO_2_ (ETCO_2_) between 35 and 45 mmHg (Avance S5 Carestation, GE Healthcare, USA). Data were manually entered on an anaesthesia record every 5 minutes for the entire duration of anaesthesia. Heart rate (HR), non‐invasive blood pressure (NiBP), respiratory rate (RR), FE'Iso, etCO_2_ and oxygen saturation (SPO₂) were continuously monitored (Avance S5 Carestation, GE Healthcare, USA). The animals were placed on the operating table in dorsal recumbency with the head lifted by a pneumatic pad at the head of the table.

In the RG, a modified RB was performed. The skin was prepared and a 22G × 50 mm Tuohy needle (Perican; BBraun, Italy) was chosen, in contrast to the approach previously reported by Chiavaccini et al. (2017). In this modified technique, the correct needle positioning was guided by seeking a slight rotation of the ocular bulb produced by the rounded tip of the Tuohy pushing against the bulbar cone, which was identified as the “rotation sign” (Lardone et al., 2024). After detecting the “rotation sign”, the needle was advanced and when it penetrated the ocular cone a loss of resistance was perceived by the operator. Subsequently, bulb repositioning was noted. Procedural success was defined as the presence of the rotation sign and/or the loss of resistance to needle advancement. An attempt was defined as inserting the needle (as described above) and removing it completely. After three consecutive attempts, the technique was considered a failure, and no further attempts were made. Furthermore, in the event of resistance to the anaesthetic injection, the procedure was terminated and classified as unsuccessful.

A mixture containing 14.6 mg/mL of lidocaine and 2 mg/mL of ropivacaine was prepared using 5.5 mL of lidocaine 2% solution (Lidocaina 2%, Ecuphar, Italy) and 2 mL of ropivacaine 0.75% (Naropina 0.75%, AstraZeneca AB, Sweden). Then a dose of 0.1 mL/kg of this mixture (which is equivalent to 1.46 mg/kg of lidocaine and 0.2 mg/kg of ropivacaine) was slowly injected. Subjects weighing >30 kg received 3 mL to avoid injecting larger volumes into the ocular cone.

The time needed to perform the block, the rotation sign, the number of attempts to perform the block, eye centralisation and time at the start of the surgery were recorded. Complications related to the locoregional technique were also recorded.

Pre‐incisional HR and mean arterial pressure (MAP) were recorded. A bolus of fentanyl 1 mcg/kg iv (Fentadon, 50 mcg/mL, Dechra, Italy) was administered as intraoperative rescue analgesia (iRA) if the HR and/or the MAP increased by ≥30% of pre‐incisional levels for at least 5 minutes (Sarotti et al., 2022). A fentanyl bolus of 1 mcg/kg iv was administered if the HR or the MAP persisted above the threshold. In the event of intraoperative movement or the presence of a brisk palpebral reflex, or when the anaesthetist perceived an increased risk of unintentional movement by the patient (increase in HR or MAP was not related to nociception), 1 mg/kg of propofol was administered iv. When the total administration of fentanyl and propofol reached three boluses, a continuous infusion of fentanyl at 5 mcg/kg/h was started. Bradycardia (HR <60 bpm, beats per minute), hypotension (defined as two or more consecutive blood pressure readings at an interval of 2.5 minutes where MAP was <60 mmHg) and other anaesthetic complications were recorded. Cisatracurium (Cisatracurio 0.2%, Mylan Pharma, Italy; 0.2 mg/kg iv) was administered intraoperatively to induce a neuromuscular block whenever the surgeon deemed it necessary to improve surgical conditions. The neuromuscular block was monitored with train‐of‐four (TOF) stimulation and acceleromyography (AMG), employing a calibrated TOF‐AMG monitor (Stimpod NMS 450X, Xavant Technology, South Africa) on one of the hindlimbs. The needle electrodes were positioned 1 cm apart laterally, with the positive electrode placed proximally and the negative electrode distally to the tibial plateau. Stimulation was conducted every 13 seconds on the peroneal nerve, and a piezoelectric crystal was placed over the dorsal aspect of the paw. When deemed necessary, antagonisation of the neuromuscular block was provided by neostigmine (Prostigmina, 0.5 mg/mL, Meda Pharma, Italy; 0.04 mg/kg iv) preceded by atropine (Atropina Solfato, 1 mg/mL, ATI, Italy; 0.02 mg/kg iv).

The start and the end of the surgery, the number of boluses of fentanyl or propofol administered, time to extubation, time to sternal recumbency and the neuromuscular blocking agent requirement were recorded.

### Blinding

The quality of recovery was evaluated by a trained operator, who was blinded to the treatment, using a score scale (Annex 1), as described by Jiménez et al. (2012). This anaesthetist was not involved in the preparation of the animal, nor was she present during the operation. She remained outside the surgical theatre throughout and only interacted with the dog in the recovery room. In addition to the fulfilment of the scale, it was permitted to provide additional commentary concerning postoperative recovery. Furthermore, all dogs underwent periocular clipping to facilitate blinding in the recovery quality assessment.

### Statistical methods

Based on the findings of a preceding pilot study, the anticipated incidences of iRA (the primary outcome measure) were 10% and 70% in the RG and C group, respectively. A minimum sample size of 18 dogs (nine per group) was calculated with a commercial software (ClinCalc.com), assuming an α error probability of 5% and a statistical power of 80%. Categorical variables are expressed as frequency and percentage; Fisher's exact test was used to evaluate frequency distribution independence between the two groups. Not normally distributed data are reported as median and range and were analysed using the Mann–Whitney *U* test. Relative risk (RR) was reported with 95% confidence intervals (CIs). The RR for iRA was the ratio of the probability of receiving fentanyl in the RB to the probability of receiving fentanyl in the C. The RRs for propofol administrations and eye rubbing during the recovery were calculated similarly. Recovery scores >2 were arbitrarily considered a bad outcome and the RR was therefore calculated by dividing the probability of having a recovery score >2 in the RB by the probability of having a recovery score higher than 2 in the C.

Statistical analysis was performed using MedCalc Software for Windows version 12.5 (MedCalcSoftware, Ltd., Belgium). Statistical significance was set at 5%.

## RESULTS

The study population was 18 privately owned dogs; all met the inclusion criteria. Nine dogs were allocated to the RB, 9 to the C. The patient demographics and surgical procedures are reported in Tables 1 and 2, respectively. The median time to perform the locoregional block was 30 seconds (range, 20 to 50), with the first attempt for each block being successful. The time between the end of surgery and extubation and the time between the end of surgery and sternal recumbency were similar for both > groups (Table 3). Table 4 presents the intraoperative complications and data (median HR and median MAP) which did not differ between groups.

**Table 1:** Study population demographics

	Retrobulbar group (*n* = 9)	Controls (*n* = 9)	P‐value
Breed			
French Bulldog	3	1	
Maltese	1	1	
Pinscher	2		
Alaskan Malamute	1		
Jack Russel terrier	1		
English Setter	1	1	
Cocker spaniel		1	
English Bulldog		1	
Poodle		1	
Mixed		2	
German Hound		1	
Age – years (range)	9 (2 to 12)	8 (4 to 12)	0.475
Weight – kg (range)	7.5 (3 to 38)	6.5 (3 to 31.7)	0.737
ASA class – no. (%)	ASA I 7/9 (77.7)	ASA I 8/9 (88.8)	1
	ASA II 2/9 (22.2%)	ASA II 1/9 (11.1)	1

**Table 2:** Type of surgery

Surgery	Retrobulbar group (*n* = 9)	Controls (*n* = 9)	Total	P‐value
Corneal (total)	5	6	11	0.334
Keratectomy	1	1	2	
Keratectomy + corneal cross linking	2	1	3	
Central corneal thickness (CCT)	1	1	2	
Corneal reconstruction	1	3	4	
Endocular (total)	4	3	7	0.334
Intrascleral silicon prosthesis	2	1	3	
Luxation lens	1	1	2	
Valve implant		1	1	
Prosthesis + corneal reconstruction	1		1	

**Table 3:** Procedural data

Characteristic	Retrobulbar group (*n* = 9)	Controls (*n* = 9)	P‐value
Median time for RB – seconds (range)	30 (20 to 50)	N/A	
Attempts – no.	1 (1)	N/A	
Failures – no. (%)	0/9 (0%)	N/A	
Duration of surgery – minutes (range)	46 (23 to 105)	30 (5 to 72)	0.093
Extubation time – minutes (range)	5 (3 to 8)	6 (3 to 10)	0.178
Sternal recumbency – minutes (range)	14 (10 to 15)	10 (8 to 20)	0.151

**Table 4:** Intraoperative data

Characteristic	Retrobulbar group (*n* = 9)	Controls (*n* = 9)	P‐value
Bradycardia – no. (%)	1/9 (11.1)	0/9 (0)	N/A
Hypotension – no. (%)	1/9 (11.1)	1/9 (11.1)	N/A
Atropine – no. (%)	1/9 (11.1)	0/9 (0)	N/A
Heart rate (bpm) – median (range)	76 (59 to 101)	79 (58 to 147)	0.507
Mean arterial pressure (mmHg) – median (range)	71 (53 to 100)	68 (54 to 91)	0.859

The dogs in RG were statistically at lower risk of receiving a rescue bolus of fentanyl intraoperatively (primary outcome) [RR = 0.142; 95% CI (0.021, 0.936); P = 0.042] and being assigned a recovery score > 2 (secondary outcome) [RR = 0.058; 95% CI (0.003, 0.887); P = 0.040]. The median amount of fentanyl (mcg/kg) was statistically lower in the RG than in the C: 0 mcg/kg (range, 0 to 1) versus 2 mcg/kg (range, 0 to 8.49), respectively (P = 0.004) (Table 5).

**Table 5:** Intraoperative rescue analgesia (IRA), consumption of propofol, recovery scores and eye rubbing. Significance set at P < 0.05 (*)

Characteristic	Retrobulbar group (*n* = 9)	Controls (*n* = 9)	P‐value	Relative risk	95% Confidence intervals
IRA – no. (%)	1/9 (11.1)	7/9 (77.7)	0.042*	0.142	0.021, 0.936
Median fentanyl dose (mcg/kg) – no. (range)	0 (0 to 1)	2 (0 to 8.49)	0.004*		
Median time to first fentanyl bolus (minutes, range)	25	21 (2 to 74)	0.66		
Propofol – no. (%)	1/9 (11.1)	5/9 (55.5)	0.10	0.200	1.207, 16.578
Median propofol (mg/kg, range)	0 (0 to 2)	1 (0 to 3)	0.08		
Recovery scores >2 – no. (%)	0 (0)	8/9 (88.8)	0.040*	0.058	0.003, 0.887
Eye rubbing – no. (%)	0/9 (0)	5/9 (55.5)	0.08	0.090	0.005, 1.435

* Significance set at P < 0.05

The dogs in RG were at lower risk of receiving a bolus of propofol (secondary outcome of the study) [RR = 0.200; 95% CI (1.207, 16.578); P = 0.10] and showed fewer eye rubbing episodes (secondary outcome of the study) [RR = 0.090; 95% CI (0.005, 1.435); P = 0.08] than C albeit the differences were not statistically significant. The median propofol was 0 mg/kg (range, 0 to 2) in the RG and 1 mg/kg (range, 0 to 3) in the C (P = 0.08) (Table 5). One dog in each group required a neuromuscular blocking agent.

## DISCUSSION

This is the first study to investigate the efficacy of supratemporal RB in dogs undergoing corneal or endocular surgeries. In this sample, RB was effective in reducing the need for intraoperative rescue analgesia and improving the quality of recovery.

Our findings are consistent with those reported in children undergoing intraocular surgery. RB proved advantageous even when general anaesthesia was carried out because of non‐collaborative behaviour by the patient. RB was reported to reduce the need for rescue analgesia in the perioperative period and was found to be a safer and better alternative to systemic fentanyl in terms of lower respiratory depression, better stress‐response suppression and pain management (Yao et al., 2017; Ye et al., 2019). The findings of our study indicated a reduction in fentanyl consumption. Considering the growing interest in reducing opioid use, RB represents a better alternative to fentanyl administration (Carcamo‐Cavazos & Cannesson, 2022; Citarella et al., 2023).

None of the RG dogs tried to rub its eyes during recovery. This may suggest a good level of postoperative comfort in those receiving the block, which allowed for better recovery and reduced risk of self‐harm. Efforts to lower the risk of poor recovery should take priority by preventing self‐inflicted damage to the operated eye in ocular surgery patients.

The block is not time‐consuming to perform (around 30 seconds on average), and the approach described by Chiavaccini et al. (2017) may be safer than other techniques as regards accidental puncture of the eye bulb.

Our modified technique allowed good control of needle tip positioning: the operator can sense the needle tip pushing against the ocular cone and often when it penetrates the cone. In this procedure, the use of a Tuohy needle is of paramount importance, as the rounded tip enables the operator to perceive the consistency of different tissue layers, with relevance to the ocular cone. Upon sensing that the needle tip is in contact with the cone, the operator can be more confident when performing a peribulbar or RB injection. It is very likely that the short time for performing the block and its success rate were largely due to the feedback on tip positioning: the “rotation signs” when the tip reached the cone and maximum rotation followed by eye repositioning just as the cone was penetrated.

Ultrasound (US) imaging is useful in locoregional anaesthesia for complete control of needle tip positioning and local anaesthetic deposition. Based on the literature search, few cadaveric studies (Foster et al., 2021; Viscasillas et al., 2019) and clinical studies (Briley et al., 2024; Citarella et al., 2023) have been published on US‐guided RB block in dogs. Considering that local anaesthetic spread is predictable because it is injected within an anatomical space delimited by connective fascia and that our modified technique is guided by the ocular movements in relation to needle advancement, the RB as performed here seems a reasonable way to provide a locoregional block in a body area where use of a US‐guided technique is not always straightforward. Nevertheless, the inability to directly observe the position of the tip needle could still result in potential complications, including haemorrhage, subarachnoid injection and eye globe perforation.

The use of a Tuohy needle may lower the risk of damage to intraconal nerves and vessels. Haematoma is one of the most common complications after RB; however, a Tuohy needle may cause less vessel perforation than sharper needles (Soh et al., 2021). Furthermore, the 22G Tuohy needle is a good compromise between the round tip proper of this needle and the sharpness due to its small calibre.

We used a solution of lidocaine and ropivacaine at a final concentration of ropivacaine of 2 mg/mL. This choice was a compromise between gaining a fast onset and offset time to have the patient quickly ready for surgery and avoiding the risk of having an awake dog with an immobile, central, protruded and insensitive eye. Chin and colleagues (Chin & Almquist, 1983) reported a mean time of 4 hours for eye akinesia in humans undergoing RB with lidocaine 2% (3 mL), epinephrine and hyaluronidase. Low‐concentration ropivacaine was administered for prolonging a certain analgesic effect in the post‐operative period.

Mixing local anaesthetics is controversial in both human and veterinary medicine. While combinations of local anaesthetics have been used clinically for many years in humans, the rationale for combining the drugs remains unclear, especially in epidural and peripheral nerve blocks. It may be misleading to assume that the result achieved by mixing two different local anaesthetics for providing nerve block is the same regardless of the locoregional technique. Among the many factors that can influence block onset, duration and intensity are anatomy, tissue perfusion, total amount of drug administered and type of nerve. The nerve block resulting from a local anaesthetic injected into the retrobulbar space or into the brachial plexus can differ. To perform a RB, the local anaesthetic is injected into the ocular cone, a physically delimited anatomical space where the thin nerves are contained in a connective fascia and therefore easily and fully bathed by the drug.

This situation is similar to that of the subarachnoid space where the spinal cord is enclosed within the arachnoid meninges. In humans undergoing retrobulbar or spinal block, the addition of lidocaine to bupivacaine produced different results compared the administration a drug alone: faster onset and longer duration in the earlier technique; the same onset, but again a longer duration of the block in the latter (Vettese & Breslin, 1985; Yazicioglu et al., 2013). We found no studies that employed the identical local anaesthetic mixture utilised in this investigation to achieve rapid onset and offset of a lidocaine nerve block, yet with a more prolonged analgesic effect in the postoperative period through the residual block of ropivacaine. As the postoperative effects of the mixture were not evaluated in this study, it is not possible to ascertain the value of adding ropivacaine.

As regards the unpredictable toxicity of a mixture of local anaesthetics, the dose of ropivacaine (0.2 mg/kg) we used was too low to be of concern. In addition, the lidocaine–ropivacaine combination has a similar pH, which makes it reasonable to assume that its pharmacokinetics may not differ considerably.

It was decided that the doses should be expressed in mL/kg instead of mg/kg, in order to facilitate simplicity. The administration of a combination of two drugs would have resulted in an unnecessarily lengthy and complex reporting of the doses (in mg/kg) for each local anaesthetic. Nevertheless, this approach may prove beneficial in routine clinical practice. The mixture (concentrated at 2 mg/mL of ropivacaine and 14.6 mg/mL of lidocaine) was administered at a dose of 0.1 mL/kg, which equates to 0.2 mg/kg of ropivacaine and 1.46 mg/kg of lidocaine.

Anaesthesia was maintained for the entire duration of surgery with a targeted FE'Iso of 1.1% in both groups. Although not statistically different, there was a tendency to require more propofol in the control group. The more stable anaesthesia in the RG meant that the block is a better compromise between depth of anaesthesia (with consequent cardiovascular depression) and nociceptive stimulation control. Moreover, anaesthetist interventions were less frequent and recovery from anaesthesia was easier to manage, making the work in the operating room safer and probably more efficient.

This study has several limitations. The data on the small retrobulbar group do not allow for speculation about the technique's real safety regarding complications such as chemosis, ecchymosis, retrobulbar haematoma, central spread of local anaesthetic, brain stem anaesthesia, ocular globe perforation and optic nerve damage. In humans, the number of life‐threatening events after regional anaesthesia techniques for eye surgery is low at 3.4 per 10,000 cases (Eke & Thompson, 1999), as major complications (Kumar & Dowd, 2008; Ripart et al., 2000). The incidence of minor complications (*e.g*. retrobulbar haematoma and haemorrhage) is 0.005% to 1.7% (Edge & Nicoll, 1993; Hamilton, 1994). While supratemporal block may have a lower incidence of ocular bulb perforation, deposition of local anaesthetic close to the optic foramen may be more likely because of needle positioning, which could theoretically increase the risk of brainstem anaesthesia. A case of brainstem anaesthesia was, in fact, reported after US‐guided RB via the supratemporal approach in a cat (Papastefanou & Rioja, 2023).

The anaesthesia protocol in this study dictated light premedication and a fixed expired fraction of isoflurane. This setting fostered the incidence of lighting of anaesthesia episodes followed by propofol administration in the control group. When the anaesthetic plane is light, iRA can be contextual with clinical signs of inadequate depth of anaesthesia. Only one retrobulbar group dog required propofol.

Corneal lesions requiring repair surgery can be caused by a variety of clinical situations depending on levels of pain and inflammation. The present sample was not stratified for such events, which may have introduced a source of error.

Intraocular pressure (IOP) was not measured. The introduction of several millilitres of local anaesthetic into the orbit would be expected to lead to a rise in IOP. Indeed, a rise in IOP has been demonstrated following peribulbar anaesthesia in humans (Bowman et al., 1996). However, a reduction in IOP has been shown following sub‐Tenon blocks, possibly due to a reduction in muscle tone (Alwitry et al., 2001). In veterinary medicine, sedation and general anaesthesia make difficult to study potential changes in IOP. Hofmeister et al. (2006) reported that the ventromedial rotation of the eye caused by propofol induction prevented accurate IOP readings in most of the dogs. Hazra et al. (2008) reported no changes in IOP in dogs anaesthetized with xylazine, ketamine and diazepam after RB and 6 and 24 hours after the surgery.

This study aimed to evaluate the intraoperative nociception and the quality of anaesthetic recovery. While the first objective was not blinded, the second was conducted by a blinded investigator. Further studies may seek to elucidate the extent to which the RB provides postoperative analgesia.

Supratemporal RB in dogs undergoing ocular surgery was easy and fast to perform, with less need for intraoperative rescue analgesia and better recovery. Further studies investigating an efficacious injectable solution are needed.

### Acknowledgment

Open access publishing facilitated by Universita degli Studi di Torino, as part of the Wiley – CRUI‐CARE agreement.

### Author contributions

**E. Lardone:** Data curation (equal); formal analysis (equal); methodology (equal); resources (equal); writing – original draft (equal); writing – review and editing (equal). **M. Crasta:** Data curation (equal). **P. C. Ostan:** Data curation (equal). **P. Gherlinzoni:** Data curation (supporting); investigation (equal). **A. Landi:** Data curation (supporting). **P. Franci:** Conceptualization (lead); data curation (lead); formal analysis (lead); investigation (lead); methodology (lead); project administration (lead); resources (lead); supervision (lead); validation (lead); writing – original draft (lead); writing – review and editing (lead).

### Conflict of interest

None of the authors of this article has a financial or personal relationship with other people or organisations that could inappropriately influence or bias the content of the paper.

## Supporting information


**Annex 1.** Recovery score scale (Jiménez et al., 2012)

## Data Availability

The original contributions presented in the study are included in the article/supplementary material, further inquiries can be directed to the corresponding author.
