# Supplementary material for: Perioperative analgesic effects of a modified supratemporal retrobulbar block in dogs undergoing corneal and endocular surgery
Source: J Small Anim Pract. 2025 Jan 12;66(5):328–34. doi: 10.1111/jsap.13829 (PMC12079311; doi:10.1111/jsap.13829)
Supplement: Supplementary file 1 — Annex 1. Recovery score scale (Jiménez et al., 2012) [file JSAP-66-328-s001.docx]

**Annex 1 –** Recovery score scale (Jiménez et al., 2012).

**1** Early- extubated, easy transition to alertness, coordinated movement; Late- alert, coordinated movement. **2** Early- fairly easy transition, holds head up, no body movement attempted; Late- holds head up, no body movement.

1. Some incoordination does not startle, generally quiet.
2. Limited muscle control, startles, may paddle or whine.
3. Uncoordinated whole body movements, startles, vocalizes.
4. Emergence delirium, thrashing, cannot be easily restrained.
